# Scorfuloderma of cheek (a cutaneous tuberculosis colliquativa cutis): Case report

**DOI:** 10.1016/j.amsu.2021.102257

**Published:** 2021-03-26

**Authors:** Ayoub Sabr, Rachid Aloua, Ouassime Kerdoud, Faiçal Slimani

**Affiliations:** a Faculty of Medicine and Pharmacy, Hassan II University of Casablanca, B.P 5696, Casablanca, Morocco; b Oral and Maxillofacial Surgery Department, CHU Ibn Rochd, B.P 2698, Casablanca, Morocco

**Keywords:** Scrofuloderma, Cutaneous tuberculosis, Extra pulmonary, Diagnosis

## Abstract

**Introduction:**

Scrofuloderma is the most common form of cutaneous tuberculosis. The facial location is unusual and requires special care.

**Discussion:**

The diagnosis of cutaneous tuberculosis is difficult due to the polymorphism of the anatomo-clinical pictures as well as the difficulty of isolating the causal agent. The combination of a range of clinical and paraclinical arguments justifies starting antibacillary treatment.

**Conclusion:**

Tuberculosis is endemic disease in our country; this endemicity is attested by the multiple cases of cutaneous tuberculosis reported in the literature.

## Highlights

• Scrofuloderma is the most common form of cutaneous tuberculosis.
• Our case is a perfect example of atypical forms of extrapulmonary tuberculosis.
• The exact diagnosis is based on the combination of clinical, histological, immunological and biological conditions.

## 1 Introduction

Tuberculosis is a chronic and contagious infectious disease that is endemic in developing countries [2,12]. It can affect all organs of the body [11,14]. Skin localization is rare and characterized by polymorphism of the anatomoclinical images and the numerous differential diagnoses [2,6,11,14]. Scrofuloderm is among the common forms of cutaneous tuberculosis often secondary to an underlying tubercular focus [2,[4], [5], [6],^8^,^11^,^14^]. It is hard to diagnose and is most often based on a series of anamnestic, clinical, histological, biological, evolutionary, and therapeutic arguments [7].

This work has been reported in line with the SCARE 2020 criteria [15].

## 2 Case report

A 28-year-old white female, with a history of pulmonary tuberculosis treated 2 years ago, presented in the outpatient department, with right cheek multiple subcutaneous nodules on the cheek. The swelling had been gradually increasing in size for the previous 6 months. There was no history of cough, fever, lack of appetite or weight loss.

**Fig. 1:**
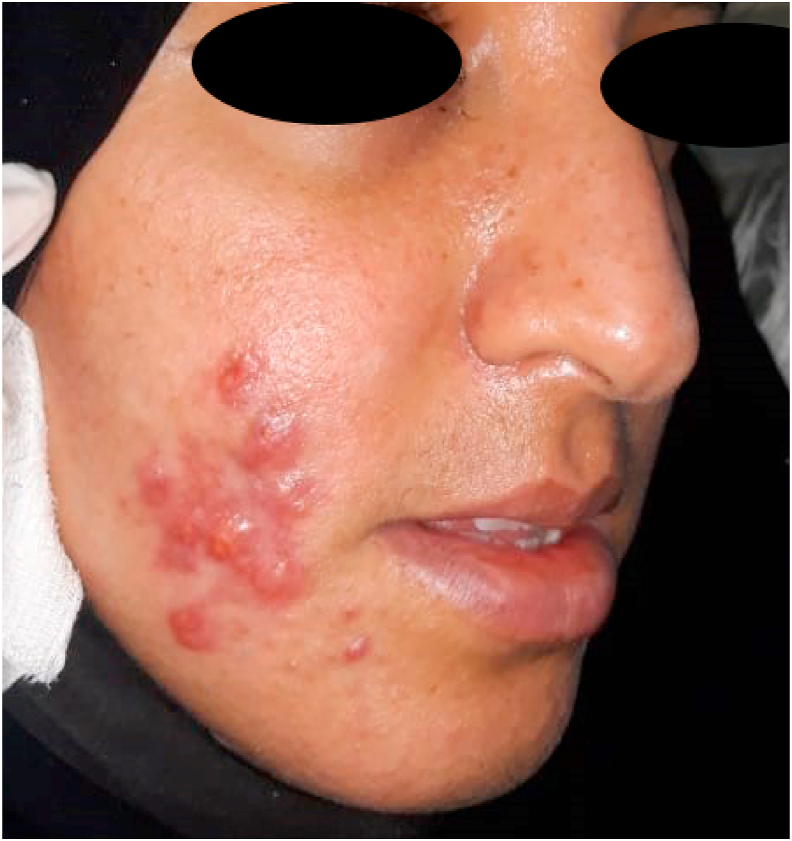
Macroscopic aspect of subcutaneous nodules.

On examination, a multiple juxtaposed straight jugular skin nodules was palpable on the right cheek, painful, mobile and smooth to the deep plane but its appears fixed to underlying bone, with pus exit on pressure (Fig. 1).

**Fig. 2:**
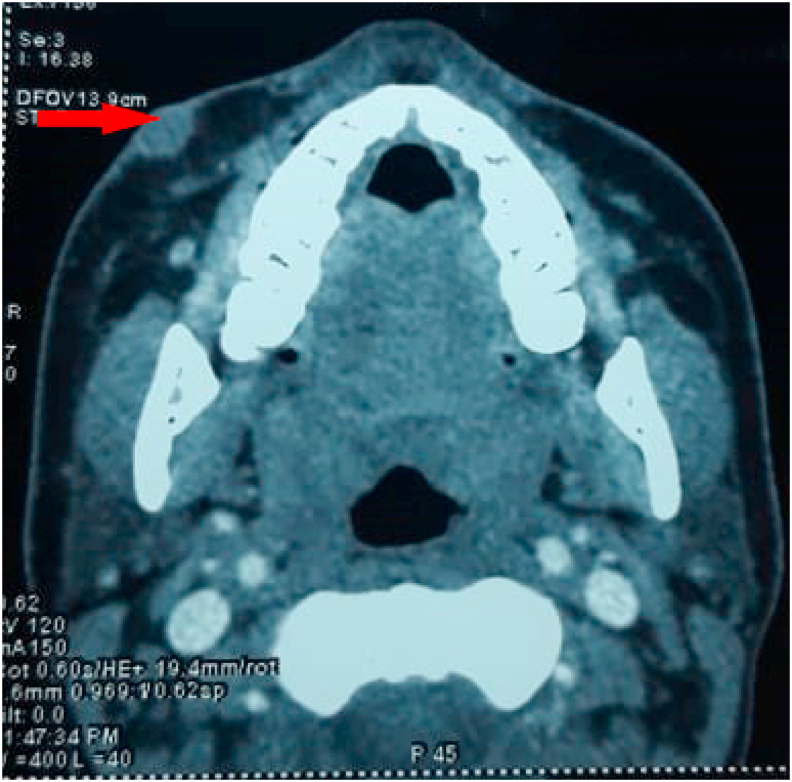
Axial section CT scan of the face, showing the soft tissue sub-cutaneous premaxillary nodules.

A facial CT was performed, which revealed well-defined, lobulated, moderately enhancing soft tissue hypodensity multiple nodular formations in the right subcutaneous premaxillary space associated with multiple bilateral cervical adenopathies (Fig. 2).

The bacteriological examination of the chessy (pus) sample did not reveal any germ on direct examination and the culture was sterile. The intradermal tuberculin reaction was negative. The investigation for other tubercular foci at a distance also came back negative.

**Fig. 3:**
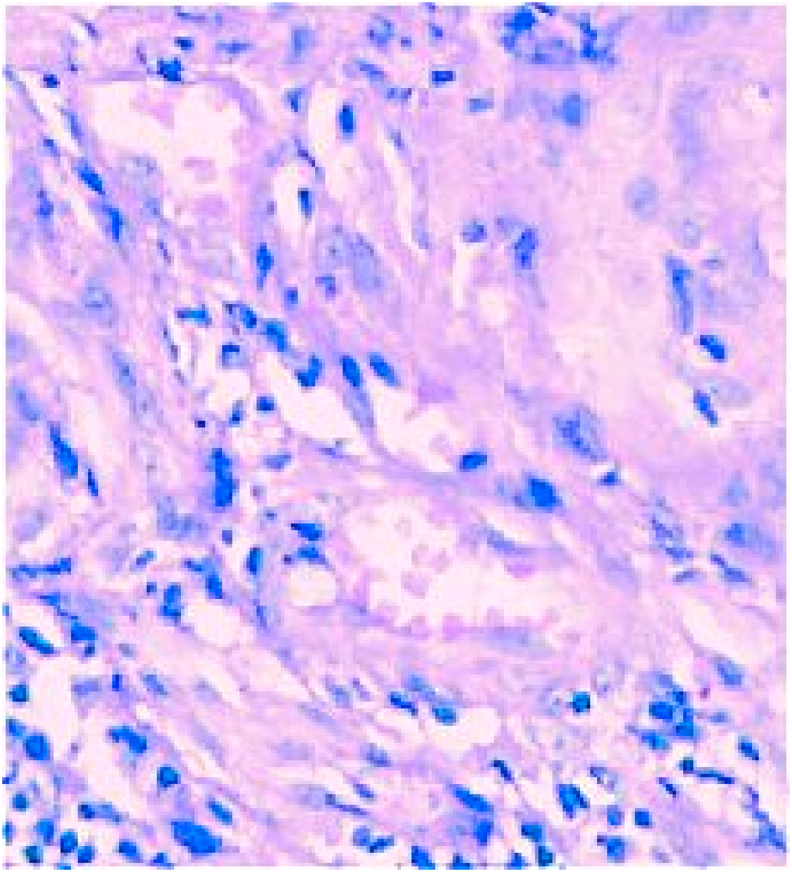
Histopathological picture of scrofuloderma with tuberculoid granuloma.

The diagnosis of Scrofuloderma was retained based on the histopathology of the lesions, which showed a tuberculoid granuloma (Fig. 3). After infectiologiste advice the patient was started on standard multidrug antitubercular therapy.

## 3 Discussion

Cutaneous tuberculosis includes all the cutaneous manifestations due to the mycobacteria of the tuberculosis complex: M. tuberculosis, M. bovis and M. africanum [1]. It is a rare localization which represents 2% of the extra pulmonary localizations of the disease (2.11).

Scrofuloderma, also known as tuberculosis colliquativa cutis, is a multibacillary form of cutaneous tuberculosis that is of interest to young adult patients from countries with tuberculosis endemicity or low to moderate immunity to the tubercle bacillus [[2], [3], [4],^9^]. It corresponds to the extension to the skin of an underlying tubercular focus (lymph node or osteoarticular) forming cold skin abscesses [2,4,5,13,14], with the cervical lymph nodes being the most involved (5,6,15). The facial location is unusual and few cases have been reported in the literature.

Confirmation of the diagnosis of scrofuloderm is difficult and is based on the comparison of a set of clinical and paraclinical data [7,9]. Clinically, in the initial stage, it manifests itself as mobile subcutaneous nodules, then these nodules soften to form painless fluctuating abscesses, secondarily the skin is perforated with the formation of depressed ulcers with purple and irregular edges, with a detached appearance, with a yellowish granular base and fistulas discharging a seroconcentrated pus, then a skin ulceration giving way to a retractile scar or keloid [2,3,14]. The search for tubercular visceral foci other than the contiguous one is essential (17). Biologically, an inflammatory syndrome is common and the intradermal tuberculin reaction is often positive. Isolation of the germ in purulent secretions or in biopsy sampling is difficult, direct examination for RABA is sometimes positive and the culture is sometimes negative [[9], [10], [11],^14^]. The most frequent histological aspect is the tuberculoid granuloma consisting of a cluster of epithelioid cells and giant Langhans cells surrounded by a mantle of mononuclear cells. The tuberculous granuloma with caseous necrosis in its centre is less frequent. Histological analysis may find non-specific inflammatory tissue, as the biopsy does not always reach the granuloma, which is sometimes deep in the hypodermis or eccentric [9,13,14]. PCR (polymerasechainreaction) is a molecular biology technique which allows rapid identification of mycobacteria and a rapid start to treatment [2,14]. The quantiferon test is much more specific for *Mycobacterium tuberculosis* than other subtypes of mycobacteria [9].

The treatment of cutaneous tuberculosis is that of a tuberculosis disease, in the immunocompetent subject, it comprises an initial phase of two months associating four antibacillary drugs: Rifampicin (10mg/kg/day) + Isoniaside (5mg/kg/day) + Pyrazinamide (20–30 mg/kg/day) + Etambutol (15–20 mg/kg/day), followed by a 2nd phase comprising a dual therapy with rifampicin and isoniaside [2,9]. The duration of treatment is extended to 9 or 12 months in immunocompromised subjects. Facial localization requires, in combination with antibacillary treatment, the prevention of retractile scars.

The various forms of tuberculosis are prevented by vaccination with the Bacille Calmette et Guérin (BCG) vaccine and by the fight against both poverty and crowding [2].

## 4 Conclusion

Cutaneous tuberculosis is an underestimated clinical form of tuberculosis which is difficult to recognise due to the polymorphism of the anatomical-clinical pictures, the challenge of detecting Koch's bacillus and the many possible differential diagnoses. The exact diagnosis of the cutaneous form of tuberculosis is based on the combination of clinical, histological, immunological and biological elements. Therapeutic management is codified and requires close monitoring to detect resistance to treatment or the appearance of side effects and long-term monitoring to detect a relapse.

## Provenance and peer review

Not commissioned, externally peer reviewed.

## Ethical approval

Written informed consent was obtained from the patient for publication of this case report and accompanying images. A copy of the written consent is available for review by the Editor-in-Chief of this journal on request.

## Sources of funding

The authors declared that this study has received no financial support.

## Author contribution

Ayoub Sabr: Corresponding author writing the paper.

Rachid Aloua: writing the paper.

Ouassime kerdoud: writing the paper (Discussion part).

Faiçal Slimani: Correction of the paper.

## Declaration of competing interestCOI

Authors of this article have no conflict or competing interests. All of the authors approved the final version of the manuscript.

## Consent

Written informed consent was obtained from the patient for publication of this case report and accompanying images. A copy of the written consent is available for review by the Editor-in-Chief of this journal on request.

## Registration of research studies

1.Name of the registry:
2.Unique Identifying number or registration ID:
3. Hyperlink to your specific registration (must be publicly accessible and will be checked):

## Guarantor

Ayoub Sabr.
